# Incremental costs of integrated PrEP provision and effective use counselling in community‐based platforms for adolescent girls and young women in South Africa: an observational study

**DOI:** 10.1002/jia2.25875

**Published:** 2022-02-07

**Authors:** Edinah Mudimu, Jack Sardinia, Sahar Momin, Andrew Medina‐Marino, Charl Bezuidenhout, Linda‐Gail Bekker, Ruanne V. Barnabas, Kathryn Peebles

**Affiliations:** ^1^ Department of Decision Sciences College of Economic and Management Sciences University of South Africa Pretoria South Africa; ^2^ Department of Health Policy and Management School of Public Health Columbia University, Columbia Mailman USA; ^3^ Research Unit Foundation for Professional Development East London South Africa; ^4^ Desmond Tutu HIV Centre Institute of Infectious Disease and Molecular Medicine University of Cape Town Cape Town South Africa; ^5^ Department of Psychiatry Perelman School of Medicine University of Pennsylvania Philadelphia Pennsylvania USA; ^6^ Department of Medicine University of Cape Town Cape Town South Africa; ^7^ Department of Global Health School of Public Health and Medicine University of Washington, Seattle Washington USA; ^8^ Department of Medicine School of Medicine University of Washington, Seattle Washington USA; ^9^ Department of Epidemiology School of Public Health University of Washington, Seattle Washington USA

**Keywords:** HIV prevention, CBCT platforms, differentiated care, PrEP effective use, micro‐costing, adherence

## Abstract

**Introduction:**

Adolescent girls and young women (AGYW) are a priority population for pre‐exposure prophylaxis (PrEP), a highly effective HIV prevention method. However, effective PrEP use among AGYW has been low. Interventions to support PrEP effective use may improve pill‐taking. Affordability of PrEP programs depends on their cost. We, therefore, evaluated the cost of community‐based PrEP with effective use counselling.

**Methods:**

Cost data from a randomized controlled trial were used to evaluate the cost of PrEP provision with effective use counselling offered to AGYW through community‐based HIV testing platforms between November 2018 and November 2019. AGYW were randomized to receive (1) group‐based community health club effective use counselling, (2) individualized effective use counselling or (3) community‐based PrEP dispensary. Task shifting of effective use counselling from nurses to trained lay counsellors was implemented in groups 1 and 2. Personnel costs were estimated from time‐and‐motion observations and staff interviews. Expenditure and ingredients‐based approaches were used to estimate costs for medical and non‐medical supplies.

**Results:**

In total, 603 AGYW initiated PrEP and accrued a total of 1280 months on PrEP. Average cost per person‐month on PrEP with group‐based community health club, individualized effective use counselling and community‐based PrEP dispensary under the Department of Health scenario were similar and high (USD $55.32, $55.65 and $55.46, respectively) due to low PrEP client volume observed in the clinical trial. Increasing client volume (scaled Department of Health scenario) reduced cost per‐person month estimates to USD $15.48, $26.40 and $13.99, respectively.

**Conclusions:**

As designed, individualized effective use counselling increased the cost of standard‐of‐care PrEP delivery by 89%, group‐based community health effective use counselling increased the cost of standard‐of‐care PrEP delivery by 11%. These estimates can inform cost‐effectiveness and budget impact analysis for PrEP provision with effective use counselling services.

## 1 INTRODUCTION

Among the HIV priority population groups [1] in South Africa, adolescent girls and young women (AGYW [16–25 years]) have the highest HIV incidence [2]. Pre‐exposure prophylaxis (PrEP) is a highly effective prevention method when used with high adherence (effective use) [3, 4]; hence, South Africa started rolling it out in 2016. However, PrEP effective use among AGYW has been sub‐optimal [5]. Additionally, there is a paucity of information on affordability and sustainability of providing the necessary PrEP delivery support services, which will ensure improved access, effective use and continuation on PrEP to achieve maximum prevention benefits [6].

Estimates of costs from current PrEP implementation research and demonstration projects that meet the needs of PrEP users in sub‐Saharan Africa are essential to serve as a foundation for cost‐effectiveness and budget impact analysis in the region. Research studies for differentiated PrEP service delivery models outside sub‐Saharan Africa have shown that delivery of PrEP in community pharmacy settings [7] and tele‐medicine‐assisted models [8] is feasible and acceptable. However, there is a lack of data on the cost of such service delivery models in sub‐Saharan Africa, which may vary depending on characteristics of the intended users [9], cost of PrEP delivery [10] and existence of other interventions [11]. PrEP cost‐effectiveness analyses in sub‐Saharan Africa have instead used indirect evidence of the cost of PrEP [12, 13, 14]. To fill this knowledge gap, evidence on the cost of delivering PrEP and effective use counselling services across varied delivery models and population groups in sub‐Saharan Africa is needed.

We, therefore, evaluated the incremental cost of community‐based PrEP provision and effective use counselling for AGYW using data collected from a randomized, controlled clinical trial.

## 2 METHODS

### 2.1 Study design

Community‐based HIV counselling and testing (CBCT) offers HIV prevention, counselling and testing services at locations outside clinic settings. CBCT centres have the benefit of being located near where people live, taking HIV testing services outside of the traditional clinical setting (usually perceived with stigma) and offering youth‐friendly services. These benefits increase the yield of linking both HIV‐negative and HIV‐positive individuals to prevention and treatment services [15]. The Community PrEP Study was established to investigate how existing CBCT platforms can be used to identify and deliver PrEP to AGYW aged 16–25 years and to determine which effective use counselling interventions are feasible, acceptable and effective. This costing study was conducted in two research CBCT sites in East London, South Africa (a rural site and an urban site). Enrolment started in November 2018 at both sites. Medically eligible (HIV negative, not pregnant and creatinine clearance > 60 ml/minute) AGYW screened for HIV potential exposure behavioural factors and interested in taking PrEP were randomized to receive (1) community‐based medication dispensary of PrEP refills (control arm – standard‐of‐care PrEP provision and monitoring in South Africa with passive adherence support), (2) group‐based health clubs effective use counselling and medication dispensary or (3) individual effective use counselling with medication dispensary. Detailed information about the control and intervention arms is available in the study protocol [16].

We will refer to the three arms as standard care, clubs and individual, respectively. Medication dispensary was done by the nurse for all participants. Trained social workers provided counselling for arms 2 and 3. PrEP initiation procedures were the same for all participants, regardless of randomization. All PrEP service delivery in the study was provided within an existing CBCT platform by CBCT HIV care staff and social workers who received training specifically for this study. Enrolled AGYW were asked to return for an orientation visit 2 weeks post‐enrolment during which test results at enrolment were reviewed and more information about PrEP uptake was shared. Thereafter, monthly follow‐up visits were scheduled for 12 months, with all follow‐up visits occurring at the same site where participants enrolled. HIV testing was done every 3 months for the duration of the study period.

Prior to enrolment, all participants provided written informed consent, which included counselling about randomization, procedures in each study group and their rights as research participants. The University of Cape Town Human Research Ethics Committee approved a waiver for guardian proxy consent for minors who opted not to involve parents via the Ministerial Consent for Non‐Therapeutic Research with Minors. The study protocol [16] was reviewed and approved by the University of Cape Town Research Ethics Committee with respect to scientific content and compliance applicable to research and human subjects’ regulations. The Community PrEP study is registered with ClinicalTrials.gov (NCT03977181) and is available at https://clinicaltrials.gov/ct2/show/NCT03977181.

### 2.2 Data sources

Cost data for PrEP delivery were collected for programmatic activities, which included (1) start‐up activities: capital inputs (clinical equipment, furniture and stationery), demand creation (posters and flyers) and initial PrEP training; and (2) ongoing activities: quarterly refresher trainings, supervision and administration (monthly reporting of PrEP uptake and continuation, and monthly PrEP accounting), overhead costs (building, utilities, fuel, maintenance and internet) and direct service delivery activities (PrEP dispensing, effective use counselling, HIV testing, syphilis testing, pregnancy testing, creatinine clearance and hepatitis B surface antigen [HBsAg] testing).

Study budgets and expense reports were utilized to estimate staff salaries for all personnel. Total and unit costs of recurrent components, which include clinical supplies, utilities, rentals, laboratory tests, medical and non‐medical supplies, were estimated using invoices, receipts, claim forms and contract agreement forms in case of services offered by contractors. Effective use counselling service costs and personnel time (nurses and social worker time spent on PrEP services) costs were estimated using data collected through time‐and‐motion observations and clinical staff interviews. Cost data for demand generation activities – posters, flyers and household visits were used to estimate the cost of getting one AGYW to be screened for PrEP provision at CBCT platforms. Costs were collected in South African Rand (ZAR) and converted to United States dollars (1 USD = 14.31 ZAR) using an average monthly market exchange rate calculated for the data collection period from 1 November 2018 to 1 November 2019 [17].

### 2.3 Cost analysis

**Figure 1:**
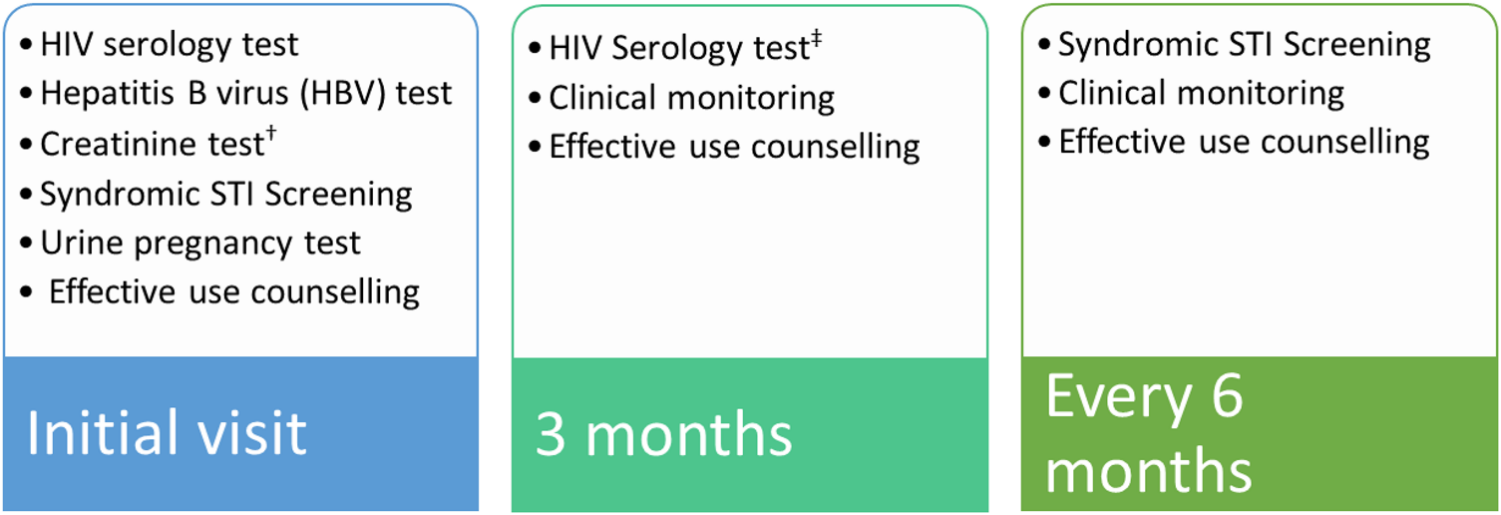
South Africa PrEP provision guidelines: graphical depiction of tests required at initiation and follow‐up visits.^†^ Creatinine test is recommended at PrEP initiation but not essential in well individuals under the age of 40. It should, however, be performed at PrEP initiation and repeated at months 6 and 12 in clients with co‐morbidities, above 40 years, pregnant women or on concomitant medication [21].^‡^ HIV serology test is required at initiation and at least every 3 months.

We estimated the average cost per person‐month of PrEP medications dispensed for each service delivery model from a provider perspective following the Global Health Cost Consortium Reference Case (GHCC) [18] and PrEP Costing guidelines outlined by the Optimizing Prevention Technology Introduction on Schedule (OPTIONS) Consortium [19]. The average cost per person‐month is defined as the total annual cost divided by the total number of person months of PrEP coverage. Costs for the three arms were estimated (1) as implemented in the study (as‐implemented scenario), (2) as would be incurred if the South African Department of Health (DoH) PrEP guidelines are followed at initiation and follow‐up visits (DoH scenario; Figure 1) [1, 20,] and (3) as would be incurred by DoH if the program were at‐scale (scaled‐DoH scenario).

Costs are either fixed or variable (Table 1). Fixed costs include capital costs of newly purchased goods for PrEP provision, demand creation, facility overheads, supervisory personnel supporting PrEP delivery, administrative supplies (PrEP evaluation and monitoring tools, such as client files, folders, requisition books, registries, diaries, etc.) and training (initial and refresher). Variable costs include personnel time (nurses and social workers time spent on PrEP services), PrEP medication and medical supplies (laboratory testing and other miscellaneous consumable supplies).

**Table 1:** Cost categories, assumptions and data sources for PrEP provision in community‐based HIV counselling and testing (CBCT) platforms

Cost category	Assumptions	Data source
**Fixed costs**		
Capital costs	Limited to PrEP‐specific costs, which include the purchase of a new fridge to store blood samples, filing cabinets, tents and chairs for each research centre, as required.	Study invoices
Training	Includes the cost of refreshments, the cost of training materials and the value of the trainer's labour.	Study budgets and GLs, project expense reports
Demand creation	Includes the value of time of study personnel to develop demand creation materials and the cost of printed posters and flyers.	Study budgets and GLs
Personnel	Includes supervisory personnel time supporting PrEP delivery.	Study budgets and GLs
Overhead costs	Includes utilities (water and electricity), rentals, maintenance and repairs.	Organization's invoices
Admin supplies	Includes the cost of PrEP monitoring and evaluation tools, such as materials to open and maintain PrEP client files, folders, lab requisition books, stamp, logbooks, registries, diaries and airtime to contact PrEP clients.	
**Variable costs**		
Personnel	Calculated as PrEP service delivery time and other PrEP‐related activities. The latter include PrEP individualized effective use counselling and group‐based effective use counselling sessions.	Study budgets and GLs
PrEP medication	Includes the cost of PrEP drug, as well as central storage and distribution fees and a one‐time PrEP importation fee.	Study budgets and GLs
Medical supplies	Includes the cost laboratory testing (pregnancy test, hepatitis B test, HIV test and creatinine test) and cost of miscellaneous supplies (e.g. gloves and swabs) required for the tests.	NHLS and testing contract

Abbreviations: GLs, general ledgers; NHLS, National Health Laboratory Services; PrEP, pre‐exposure prophylaxis.

We computed personnel time at PrEP initiation visits, follow‐up visits and effective use counselling sessions for each service delivery model by observing the total time needed and removing both waiting time for participants and time dedicated to research activities. Visits were classified as monthly refill visits (occurring at months 1, 2, 4, 5, 7, 8, 10 and 11, and including PrEP refill and effective use counselling) and quarterly refill visits (occurring at months 3, 6, 9 and 12, and including PrEP refill, effective use counselling, blood tests and a pregnancy test). with all follow‐up visits occurring at the same site where participants enrolled. We averaged observed times to estimate personnel time spent on PrEP delivery activities. Clinical personnel cost was estimated by multiplying the observed average time spent on each PrEP delivery activity by the personnel cost per minute (calculated by using the annual staff salary, including allowances, and assuming that each staff worked 8 hours per day for 260 days, excluding standard holidays and leave, a year). Overhead costs for PrEP provision through CBCT platforms were proportionally allocated according to the fraction of total activities (initiation, monthly and quarterly follow‐up visits) that were dedicated to PrEP provision. PrEP medication costs per month for the as‐implemented scenario include the commodity cost as well as importation and central storage and distribution fees. DoH PrEP medication cost per month was obtained from recently published data [6]. Total medication costs were determined by multiplying the cost per month supply by the number of person‐months on PrEP. Total costs for HIV testing, syphilis testing, pregnancy testing and laboratory testing were estimated as the product of the unit cost of each test, including clinical supplies, and number of tests done during the costing period.

Because PrEP delivery and effective use support were provided within existing CBCT platforms, we assume that select fixed costs (demand creation, capital and overhead) under the DoH scenario and scaled‐DoH scenario were equal to those in the as‐implemented scenario. The other cost drivers for the DoH scenario and scaled‐DoH scenario are based on government‐specific costs, that is we used (1) unit cost for tests as listed by the National Health Laboratory Services [22]; (2) salaries and basic allowances for staff based on the South African salary wage scale [23]; (3) PrEP drug cost as published by Meyer‐Rath et al. [6]; (4) pregnancy testing is at no cost in our DoH scenarios since it occurs through the family planning department and only done at initiation; and (5) required tests as per DoH guidelines (Figure 1). We excluded creatinine testing in our DoH and scaled‐DoH scenario analysis since it is not essential for AGYW [21]. A detailed description of items included in each costing category is in Table 1 and Table 2. We further assumed that the number of clients for each type of visit who could be seen per day for the DoH scenario is the same as in the as‐implemented scenario. For the scaled‐DOH scenario, we estimated the number of clients for each type of visit using the time spent by health personnel and the proportion of the type of visit observed during the study year for each service delivery model.

**Table 2:** Assumptions for staff annual salaries, unit cost for tests (2020 United States dollars), PrEP services activities and number of clients for the as‐implemented scenario and DoH scenarios

	As‐implemented scenario		DoH scenarios	
Job title	Annual salary	Hourly salary^a^	Annual salary	Hourly salary^a^
Nurse	25,474.91	12.25	13,876.22	6.67
Health club counsellor	5963.09	2.87	2318.16	1.11
Individual effective use counsellor	4587.16	2.21	2318.16	1.11

Laboratory tests		Unit cost^b^		Unit cost^c^
Medical supplies		0.08		0.08
HIV serology test		13.35		4.44
Hepatitis B virus (HBV) test		13.75		10.12
Creatinine test		3.44		2.44
Pregnancy test (urine BhCG)		0.34		0.00
STI screening		42.17		2.64
Hepatitis B vaccination		7.62		1.68
TFV‐DP level test		87.62		0.00
PrEP drug cost		11.00		4.72

PrEP service activities		
HIV test	Initiation, month 1 and every 3 months thereafter	Initiation, month 1 and every 3 months thereafter
Creatinine test	Initiation, months 3 and 12	Creatinine test is recommended at PrEP initiation but not essential in well individuals under the age of 40. It should, however, be performed at PrEP initiation and repeated at months 6 and 12 in clients with co‐morbidities, above 40 years, pregnant women or on concomitant medication^d^
Pregnancy test	Initiation, months 3, 6, 9 and 12	Initiation only, subsequent tests through family planning department
Hepatitis B test	Initiation	Initiation
Number of clients	As observed during the study	DoH – same as in the as‐implemented scenario Scaled‐DoH – estimated using the time spent by health personnel and the proportion of the type of visit observed during the study year for each service delivery model. Detailed calculations are in Data S1 clients tab

Abbreviations: BcHG, beta‐human chorionic gonadotropin; DoH, Department of Health; PrEP, pre‐exposure prophylaxis; TFV‐DP, tenofovir‐diphosphate.

^a^ Hourly salary for each profession is estimated by dividing mean estimated annual salary by 2080 (this excludes standard holidays in a year).

^b^ Unit cost for as implemented scenario obtained from study receipts and invoices.

^c^ Unit costs for the as implemented scenario obtained from the National Health Laboratory Service Price List [22] and from [6].

^d^ PrEP 2020 guidelines [21].

All visits by clients who never initiated PrEP were excluded from the cost estimation. The annualized cost of capital goods is discounted at a standard rate of 3% [0–5% range] [24]. We assumed a useful life of 3 years for all capital items. Cost data analysis was done using Excel (version 1807, Microsoft, Redmond, WA).

## 3 RESULTS

**Figure 2:**
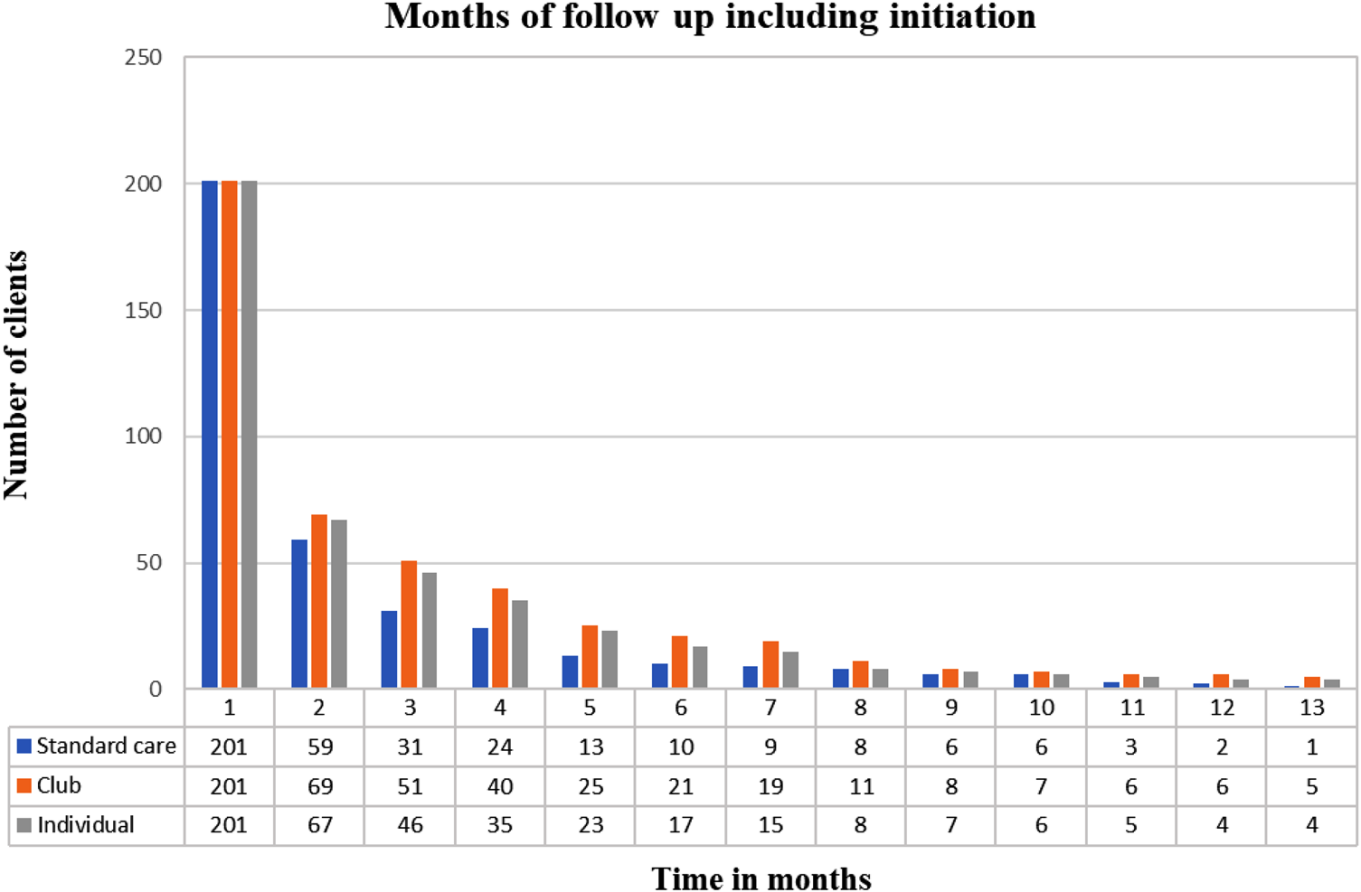
Months of follow‐up for standard care, club and individual service delivery models.

A total of 603 AGYW initiated PrEP, accruing 1280 total months (median duration of PrEP was 1 month, IQR:1, 2) of PrEP between 1 November 2018 and 1 November 2019 (Figure 2 shows months of follow up). Of those who initiated PrEP, 51% were between the ages of 16 and 18 years. We completed 50 time‐and‐motion observations, including 9 initiation, 15 standard care, 8 club and 18 individual visits. The cost to get one AGYW at the door (demand creation) is approximately $7.17. Unfortunately, the study did not track the number of AGYW to whom PrEP was offered but did not enrol. Hence, we cannot include the additional cost of personnel time for unsuccessful recruitment in the as‐implemented scenario. However, because there is no similar recruitment as part of the standard of care in South Africa, there is no impact on the cost estimates from the DOH and scaled‐DOH scenarios.

To estimate the number of clients for each type of visit who could be seen per day for the scaled‐DOH scenario, we used the time spent by health personnel and the proportion of the type of visit observed during the study year for each service delivery model (Table 3). We assume that only one of the three models will be implemented; hence, the numbers that can be accommodated by each model is not affected by staff availability neither is there a need for staff prioritization to the service delivery models. Of visits included during the 1‐year costing period for the standard care service delivery model, 54% were initiation visits, 35% were monthly refill visits and 11% were quarterly refill visits. The club service delivery model had 63% initiation visits, 27% monthly refill visits and 10% quarterly refill visits. For the individual service delivery model, 46% of visits were for initiation, 40% for monthly PrEP refills and 14% for quarterly visits. For detailed calculation, see “clients” tab in Data S1.

**Table 3:** Time spent (minutes) by health personnel and the maximum number of clients who can be accommodated for each visit type per day

Time spent^a^ in minutes for each type of visit per day						
	Standard care		Club		Individual	
Visit type	Social worker	Nurse	Social worker	Nurse	Social worker	Nurse
Initiation	19	34	19	34	19	34
Monthly	1	6	41	2	28	2
Quarterly	1	16	37	19	27	21
Total time	21	55	97	55	75	57

Maximum number of clients for each type of visit per day			
Visit type	Standard care	Club	Individual
Initiation	7	8	11
Monthly visit	29	3^b^	6
Quarterly	3	1^b^	2
Total utilized time per day (minute)			
Social worker	154	314	450
Nurse	450	447	428

^a^ Time spent by staff – standard care service model is per client. In the club service model, time spent by the social worker is per group and by the nurse is per client. In the individualized service model, time spent by staff is per client.

^b^ Number of group visits per day. Group size for monthly and quarterly visits is 7; hence, total number of clients seen is obtained by multiplying 7 by number of groups.

In all service delivery models, the nurse and social worker work in series (i.e. not in parallel). The maximum number of clients who can be served per day is, therefore, constrained by the health worker with the total maximum time for the service (initiation, monthly and quarterly visits) offered. For example, in standard care service delivery model, we use nurses’ time (total time required by a nurse is 55 minutes and social worker is 21 minutes) to determine the maximum number of clients who can be served per day.

The number of clients who can be served in a day for each service delivery model is shown in Table 3. For the club service delivery model, we determined the maximum group size of clients using the time a nurse needs to attend to all clients who attend group sessions. Detailed calculations can be found in Data S1 in the “clients” tab of the spreadsheet.

### 3.1 Personnel time

PrEP initiation visits required 51 minutes (IQR: 46, 63) of staff time, with clinical consultation accounting for approximately 63% of the total time, while the remaining time was spent with social workers. Scheduling calls and sending a text message for effective use counselling and medication pick‐up reminders to participants required 2 minutes per participant per visit (IQR: 1, 3) of social workers’ time.

A median of 6 minutes (IQR: 5, 9) was required for a monthly standard care refill visit and 17 minutes (IQR: 15, 21) for a quarterly standard care visit. In both standard care visits, less than one tenth of the time was spent with social workers. The time required for club monthly visits had a median of 43 minutes (IQR: 31, 67), and 57 minutes (IQR: 47, 62) were required for quarterly visits. There was considerable variation in the duration of monthly refill visits because the duration of effective use counselling sessions depended on the extent to which young women spoke and interacted during the session. Monthly individual refill visits were a median of 30 minutes (IQR: 17, 34), with most services (25 minutes, 96%) provided by a social worker. Individual quarterly visits required 46 minutes (IQR: 36, 55), with 56% of the services provided by social workers.

### 3.2 Costs of PrEP provision

The total annual PrEP delivery cost in the as‐implemented scenario was similar across the three service delivery models (standard care: $135,314, club: $135,143 and individual: $135,791) (Table 4). The total annual cost per service delivery model decreases by 46% for the DoH scenario (standard care: $70,994.44, club: $70,811.3 and individual: $71,227.59). The cost per person‐month for each service delivery model under the as‐implemented scenario is standard care: $105.71, club: $105.58 and individual: $106.09. In the DoH scenario, it is $55.46, $55.32 and $55.65 for the standard care, club and individual service delivery models, respectively. The main drivers of the total annual cost across all service delivery models in the as‐implemented scenario were fixed personnel costs (40%), followed by fixed administrative costs (18%) and PrEP medication costs (10%). In the DoH scenario, the main drivers were fixed personnel costs (57%), followed by PrEP medication costs and HBsAg testing costs at 8% each, with demand creation contributing 6% to the total annual cost.

**Table 4:** Estimated annual total cost and unit cost per person month of PrEP in United States dollars (US$) for the as‐implemented scenario, DoH scenario and the scaled‐DOH scenario

	As‐implemented scenario						DoH scenario						Scaled‐DoH scenario					
	Standard care		Club		Individual		Standard care		Club		Individual		Standard care		Club		Individual	
Category	Annual cost	Unit cost	Annual cost	Unit cost	Annual cost	Unit cost	Annual cost	Unit cost	Annual cost	Unit cost	Annual cost	Unit cost	Annual cost	Unit cost	Annual cost	Unit cost	Annual cost	Unit cost
**Fixed costs**																		
Capital	2295.13	1.79	2295.13	1.79	2295.13	1.79	2295.13	1.79	2295.13	1.79	2295.13	1.79	2295.13	0.23	2295.13	0.25	2295.13	0.45
Training	1659.60	1.30	1659.60	1.30	1659.60	1.30	806.12	0.63	806.12	0.63	806.12	0.63	806.12	0.08	806.12	0.09	806.12	0.16
Demand creation	4365.73	3.41	4365.73	3.41	4365.73	3.41	4365.73	3.41	4365.73	3.41	4365.73	3.41	4365.73	0.43	4365.73	0.47	4365.73	0.86
Personnel fixed	54,468.70	42.55	54,468.70	42.55	54,468.70	42.55	41,387.17	32.33	41,387.17	32.33	41,387.17	32.33	41,387.17	4.07	41,387.17	4.42	41,387.17	8.16
Overhead	6822.96	5.33	6822.96	5.33	6822.96	5.33	2699.15	2.11	2699.15	2.11	2699.15	2.11	2699.15	0.27	2699.15	0.29	2699.15	0.53
Admin (supplies), fixed	24,420.67	19.08	24,420.67	19.08	24,420.67	19.08	631.92	0.49	631.92	0.49	631.92	0.49	631.92	0.06	631.92	0.07	631.92	0.12
**Variable Costs**																		
Personnel, variable	5756.74	4.50	5586.14	4.36	6234.12	4.87	3118.46	2.44	2935.95	2.29	3351.61	2.62	11,163.84	1.10	9625.73	1.03	13,778.66	2.72
PrEP (supplies), variable	14,080.41	11.00	14,080.41	11.00	14,080.41	11.00	6047.25	4.72	6047.25	4.72	6047.25	4.72	48,052.55	4.72	44,220.50	4.72	23,949.76	4.72
Miscellaneous medical supplies (gloves and swabs)	105.58	0.08	105.58	0.08	105.58	0.08	105.58	0.08	105.58	0.08	105.58	0.08	508.27	0.05	491.74	0.05	341.14	0.07
HIV serology test	10,336.37	8.08	10,336.37	8.08	10,336.37	8.08	3434.27	2.68	3434.27	2.68	3434.27	2.68	11,726.97	1.15	17,304.48	1.85	15,088.74	2.98
HBsAg test	8293.07	6.48	8293.07	6.48	8293.07	6.48	6103.65	4.77	6103.65	4.77	6103.65	4.77	18,705.92	1.84	21,054.06	2.25	28,479.09	5.62
Creatinine test	2449.28	1.91	2449.28	1.91	2449.28	1.91	–	–	–	–	–	–	–	–	–	–	–	–
Pregnancy test	259.76	0.20	259.76	0.20	259.76	0.20	–	–	–	–	–	–	–	–	–		–	–
Total cost and cost per person month of PrEP	135,314	105.74	135,143.42	105.61	135,791.40	106.09	70,994.44	55.46	70,811.93	55.32	71,227.59	55.65	142,342.77	13.99	144,881.73	15.48	133,822.61	26.40

Abbreviations: DoH, Department of Health; PrEP, pre‐exposure prophylaxis.

In the scaled‐DoH scenario, the standard care service delivery model had the lowest cost per person‐month at $13.99. The club effective use service delivery model had a cost per person‐month of $15.48, which is 11% higher than the standard care service delivery model. Individual service delivery model was the most expensive service delivery model, with a cost per person‐month of $26.40. The individual service delivery model could accommodate 5069 person‐months of PrEP, while the standard care and club service delivery models were able to provide approximately double the number of individualized person‐months of PrEP coverage (10,171 and 9360, respectively), given the lack of staffing time for effective use counselling in the standard care service delivery model and having group effective use counselling sessions for the club service delivery model. The main driver of cost in the standard care service delivery model is PrEP medication (34%), followed by fixed personnel costs (29%), with a similar pattern for the club service delivery model (PrEP medication: 31%; fixed personnel costs: 29%). A flip in the main drivers of cost was observed in the individual service delivery model, with fixed personnel costs (31%) accounting for the largest proportion of costs, followed by PrEP medication costs at 18%.

## 4 DISCUSSION

In this micro‐costing study, we estimated that the cost to DoH of at‐scale PrEP provision integrated with group‐based community health clubs effective use counselling for AGYW resulted in a minimal increase of 11% compared to the cost of standard‐of care PrEP provision, while providing individualized effective use counselling resulted in an increase of 89%. PrEP medication accounted for the largest proportion of the cost per person‐month of PrEP, at 31%, consistent with other PrEP costing studies [9, 25]. Under the DoH scenario, added costs on per‐person month PrEP cost of $41, $40 and $29 for standard care, club and individual service delivery models, respectively, came from fixed personnel costs due to the low volume of participants enrolled for the study.

The low volume of participants also resulted in similar estimates of the cost per‐person month of PrEP in the as‐implemented scenario and DoH scenarios. Conversely, in the scaled DoH scenario, we assume that PrEP uptake will increase as community knowledge of PrEP expands, and the concomitant increase in PrEP client volume substantially reduces the cost per person‐month estimates. Critically, evidence on the effectiveness of these strategies is needed to assess cost‐effectiveness and to guide the choice between these approaches. The costly service delivery models can be designed differently to cost less while at the same time maintaining their effectiveness if they are found not to be cost‐effective.

The cost per person‐month estimates presented here were obtained from a study carried out in existing CBCT centres with trained and dedicated staff to perform effective use counselling and collect required samples for clinical tests. While CBCT centres do not exist in all parts of South Africa [26], these results can inform a comparison of costs between standard care and individual or club effective use counselling strategies.

Among the DoH‐prescribed tests, HIV serology ($4.44) and HBsAg ($10.12) tests were the most expensive. These costs highlight the need for negotiated reduced costs. For example, HIV self‐test kits are now relatively low cost ($2) as a result of a buydown agreement negotiated by the Gates Foundation [27]. The new South Africa PrEP guidelines [20] recommend a minimalist approach to laboratory monitoring to ensure feasible and affordable scale‐up of PrEP provision. The minimalist approach is supported by evidence from studies which have shown that a reduction in the frequency of creatinine testing for individuals without specific risk factors is safe [28]. Additionally, as high numbers of PrEP clients drop out before the first follow‐up visit [29], deferring creatinine testing to the first follow‐up visit has the potential to reduce laboratory costs [23].

We excluded etiologic sexually transmitted infection (STI) testing from our cost estimates since it is not part of the standard‐of‐care of PrEP provision in South Africa. However, results from PrEP demonstration projects have reported high prevalence of STIs among young African women [5, 28], the presence of which increases potential exposure to HIV acquisition [30, 31]. To address the high burden of STIs in young African women, etiologic STI testing should be integrated with the PrEP package. Cheap, point‐of‐care STI tests are needed to meet this goal.

The lack of data describing the cost of real‐world PrEP implementation programmes has contributed to wide variation in the results of PrEP budget impact and cost‐effectiveness analyses [9, 13]. Meyer‐Rath et al. [6] used an ingredient‐based approach based on assumptions, expert opinion and available literature to estimate the cost‐per‐person‐month on PrEP in South Africa and obtained an estimate of approximately US$11, excluding effective use counselling. Our study provides important unit cost data on integrated PrEP provision and effective use counselling for AGYW that may be utilized in budget impact and cost‐effectiveness analysis.

## 5 CONCLUSIONS

Individualized effective use counselling increased the cost of standard‐of‐care PrEP delivery by 89%, while group‐based community health effective use counselling clubs increased the cost of standard‐of‐care PrEP delivery by 11%. These estimates may be useful to policy makers in planning PrEP implementation and can inform cost‐effectiveness and budget impact analysis for PrEP provision with effective use counselling services in CBCT platforms.

## FUNDING

This publication was made possible through a grant from the National Institute for Mental Health (NIMH), U.S. National Institutes of Health (NIH) under award number R01MH114648 to Professors AMM and LGB. The funder had no role in the design of the study and the collection and analysis of the data and in writing the manuscript.

## COMPETING INTERESTS

RVB has received conference abstract support from Regeneron Pharmaceuticals outside the submitted work. For the remaining authors, none were declared.

## AUTHOR CONTRIBUTIONS

EM led conception of the manuscript, design as well as drafting the manuscript, data analysis and interpretation of results with contributions from KP and RVB. JS and SM contributed to the acquisition of the data, analysis, and interpretation of the data and critical revision of the manuscript. LGB, AMM and CB contributed in the conception and design and critical revision of the manuscript. All authors reviewed and approved the final version of the manuscript.

## Supporting information


**Data S1**: Data – cost calculations spreadsheet

## Data Availability

The data that supports the findings of this study are available in the supplementary material of this article.
